# Splenic Marginal Zone Lymphoma With Histological Transformation to Hodgkin’s Lymphoma

**DOI:** 10.7759/cureus.26738

**Published:** 2022-07-11

**Authors:** Sindhusha Veeraballi, Noreen Mirza, Zaineb Khawar, Hamid Shaaban

**Affiliations:** 1 Internal Medicine, Saint Michael's Medical Center, Newark, USA; 2 Internal Medicine, St. George's University School of Medicine, Newark, USA; 3 Hematology/Oncology, Saint Michael's Medical Center, Newark, USA

**Keywords:** nodular subtype hodgkin's lymphoma, cancer prognosis, tumor transformation, hodgkin's lymphoma, marginal zone lymphoma

## Abstract

Marginal zone lymphoma (MZL) is a rare, slow-growing/indolent B cell lymphoid neoplasm accounting for 10.5% to 11.8% of all B cell lymphomas. MZL originates from the mature B lymphocytes, which are usually present in the marginal zone of the lymphoid follicle. Histological transformation (HT) is defined as sheets of large cells arising in an indolent lymphoma with morphological and immunophenotypic changes suggestive of a high-grade lymphoma such as Hodgkin's lymphoma, diffuse large B cell lymphoma (DLBCL), or Burkitt lymphoma. The median time of transformation ranges from one year to 15 years following the initial diagnosis of MZL. Studies reported that the deletion of TP53 and 7q and mutations in NOTCH2 are commonly associated with HT in MZL. This case report outlines the rare happening of an MZL transformation into a nodular subtype of Hodgkin's lymphoma in a 56-year-old female, which prompted further investigations and a different therapeutic approach. By reporting this case, we emphasize that HT changes the natural history and significantly affects the overall survival of patients with MZL. Hence, it is necessary to get a core needle or excisional biopsy whenever there is a clinical suspicion of HT in MZL for early diagnosis and a better therapeutic approach.

## Introduction

Marginal zone lymphoma (MZL) is a rare, slow-growing/indolent B cell lymphoid neoplasm accounting for 10.5% to 11.8% of all B cell lymphomas and 6% to 9% of all non-Hodgkin's lymphomas [1,2]. MZL originates from the mature B lymphocytes, which are usually present in the marginal zone of the lymphoid follicle, and includes three subtypes, i.e., nodal, splenic, and mucosal-associated marginal cell lymphoma (MALT). The histologic transformation of a low-grade lymphoma to a more aggressive or high-grade lymphoma is an uncommon event that has been documented to occur in any subtype of MZL [1,2]. Overall, the rate of transformation ranges from 3% to 20% for MZL in a median time of one to 15 years. However, despite the lack of research on the histologic transformation of MZL to Hodgkin's lymphoma, it is clear that histologic transformation is associated with a significant increase in morbidity and mortality [3]. Herein, we present a rare phenomenon, i.e., the histological transformation (HT) of MZL into the nodular sclerosis subtype of Hodgkin's lymphoma.

## Case presentation

A 56-year-old lady with a past medical history of Parkinson's disease and diabetes first presented to our hematology-oncology clinic in 2018 with B symptoms like fever, night sweats, fatigue, and a 15-pound weight loss in two months with no recent travel outside the United States. She denied having any cough, shortness of breath, chest discomfort, joint pains, rash, and any gastrointestinal symptoms. Physical examination revealed no palpable lymphadenopathy. CT scan of chest, abdomen, and pelvis revealed clear lungs, but splenomegaly, enlarged splenic hilar lymph nodes (2.4 x 3.2 cm), and para-aortic lymph nodes were noted. Cytomegalovirus (CMV), HIV, hepatitis, and Epstein-Barr virus (EBV) IgM were negative. Perisplenic excisional lymph node biopsy revealed distortion of lymph node architecture by a diffuse and vaguely nodular proliferation of small lymphocytes. The lymphocytes exhibited mild polymorphisms, mildly irregular nuclear contours, and often moderate amounts of clear to amphophilic cytoplasm. Scattered immunoblasts and occasional polykaryocytes were noted. Flow cytometry showed a monoclonal kappa and CD5-positive B cell population. CD23 was not expressed. Immunohistochemistry analysis revealed a dominant/diffuse B cell population positive for CD20, PAX5, and BCL2. The cells were negative for CD5, CD10, CD23, CD43, cyclin D1, BCL6, and immunoglobulin D (IgD). The staining of Ki-67 was positive in 20-50% of cells. Multiple myeloma oncogene-1 (MUM1)-positive plasma cells were rare and polytypic. Fluorescence in situ hybridization (FISH) analysis was performed and was negative for CCND1/IGH rearrangement. The overall features were most consistent with a splenic MZL. Blood work at that time revealed elevated lactate dehydrogenase (LDH) of 532 IU/L. She was diagnosed with splenic MZL with 1% of bone marrow involvement. She received four weekly doses of Rituxan with an inadequate response and was then started on chemotherapy with rituximab, cyclophosphamide, hydroxydaunorubicin hydrochloride, vincristine, and prednisone (RCHOP). She completed six cycles of RCHOP and was started on maintenance rituximab therapy. Furthermore, she then went into clinical and radiological remission.

In early 2021, she started to experience B symptoms like fevers, night sweats, and weight loss again. It was thought that she is in recurrence of MZL refractory to RCHOP. A positron emission tomography scan was done, which showed a new and progressive fluorodeoxyglucose (FDG)-avid lymphadenopathy with 1.2 x 0.7 cm FDG-avid left internal jugular lymph node, 2.4 x 2.0 cm FDG-avid para esophageal/posterior mediastinal mass, and FDG-avid lymph nodes in the splenic hilum. Also, splenomegaly with new heterogeneous areas of FDG avidity, likely representing tumor infiltration of the spleen, was found. The Deauville score at the time was 5. There was a concern for transformation, as FDG avidity was somewhat higher than expected for MZL. Laparoscopic splenic hilar node biopsy was consistent with Hodgkin's lymphoma stage IIIB, nodular sclerosis subtype. Microscopy showed fibrotic tissue with a vaguely nodular architecture. Scattered small lymphocytes and histiocytes were present, along with scattered large atypical cells. The lymph node specimen showed rare CD30+ atypical cells in a lymphohistiocytic background. Immunohistochemical staining showed that the large atypical cells were positive for CD30, CD14, dim PAX5, MUM1, and Ki-67. They were negative for CD20 and CD45. Bone marrow biopsy was significant for moderately hypercellular marrow for age (70%) with trilineage hematopoiesis. There was no evidence of classical Hodgkin’s lymphoma. She was then started on brentuximab vedotin, doxorubicin, vinblastine, and dacarbazine (AAVD) chemotherapy for which she is showing a good response.

## Discussion

The incidence of MZL is predominant in the lymphatic tissue, which is exposed to external antigens continuously, like the spleen and mesenteric lymph nodes. Being a metalworker is a risk factor for nodal MZL, asthma and the use of hair dye are risk factors for splenic MZL, whereas infectious agents and autoimmune disorders are risk factors for extranodal MZL [4]. The pathology and natural history of MZL are poorly understood due to the rarity of the case, diverse clinical presentation, and disease heterogeneity [4]. HT is defined as sheets of large cells arising in an indolent lymphoma with morphological and immunophenotypic changes suggestive of a high-grade lymphoma such as Hodgkin's lymphoma, diffuse large B cell lymphoma (DLBCL), or Burkitt lymphoma [3,5]. The incidence of HT increases every year with a frequency of about 2.4% per year, 5% at five years, and 10% at 12 years, and is relatively high in splenic MZL when compared to other subtypes. The median time of transformation ranges from one to 15 years following the initial diagnosis of MZL [3,5]. Studies reported that the deletion of TP53 and 7q and mutations in NOTCH2 are commonly associated with HT in MZL. Clinical variables such as elevated LDH, more than four nodal sites at diagnosis, CD5 expression, complex karyotype, and failure to achieve complete remission after initial treatment are also found to be the risk factors for HT [4,6]. However, the pathogenesis behind the HT of MZL is poorly understood and can be attributed to the rarity of the case. Further research has to be done for better understanding.

## Conclusions

HT changes the natural history and significantly affects the overall survival of patients with MZL. Hence, it is necessary to get a core needle or excisional biopsy whenever there is a clinical suspicion of HT in MZL for diagnosis and a better therapeutic approach. The pathogenesis behind the HT of MZL is poorly understood and can be attributed to the rarity of the case. Further research has to be done for better understanding.
